# Fetal echocardiography

**DOI:** 10.4103/0971-3026.44524

**Published:** 2009-02

**Authors:** Nitin G. Chaubal, Jyoti Chaubal

**Affiliations:** Thane Ultrasound Centre, Thane, India; 1 Jaslok Hospital and Research Centre, Mumbai, India

**Keywords:** Arch of aorta, ductus arteriosus, fetal echo, fetal echocardiography, inferior vena cava, left ventricular outflow tract, pulmonary artery, pulmonary veins, right ventricular outflow tract, superior vena cava

## Abstract

USG performed with a high-end machine, using a good cine-loop facility is extremely helpful in the diagnosis of fetal cardiac anomalies. In fetal echocardiography, the four-chamber view and the outflow-tract view are used to diagnose cardiac anomalies. The most important objective during a targeted anomaly scan is to identify those cases that need a dedicated fetal echocardiogram. Associated truncal and chromosomal anomalies need to be identified. This review shows how fetal echocardiography, apart from identifying structural defects in the fetal heart, can be used to look at rhythm abnormalities and other functional aspects of the fetal heart.

## Introduction

Congenital heart disease (CHD) is a common anomaly in newborns. Improvements in the antenatal diagnosis of cardiac anomalies have resulted in a significant reduction in neonatal morbidity and mortality.[1,2] With early diagnosis, good intranatal and postnatal care can be offered to a baby with a cardiac anomaly and the family can be prepared emotionally and financially to accept such a baby.[3]

With improvement in operator skills and the availability of high-end machines with newer imaging techniques like spatio-temporal image correlation (STIC), multiplanar imaging, X-plane, full-volume fetal echo, and tissue Doppler imaging, the pick-up rate for cardiac anomalies has increased significantly.[1]

## Incidence of CHD

The incidence of CHD is 8 per 1000 live births.[4,5] However, the incidence is much higher in the fetal population. A good number of fetuses with complex cardiac anomalies succumb in the first trimester itself, even before the cardiac anomaly is suspected; some parents opt for termination of pregnancy after the diagnosis is made in the mid-trimester; and some cardiac anomalies are progressive and end in intrauterine death. Thus, the incidence quoted above may be only the tip of the iceberg.

## Rate of recurrence[6]

Cardiac anomalies are known to cluster in families; the risk of having a child with a cardiac anomaly is as follows:

- If a previous child was born with a CHD, the probability of a subsequent child being born with a CHD is 1:20 to 1:100.
- If two previous children were born with CHD, the risk is 1:10 to 1:20.
- If the mother has CHD, the risk is as high as 1:5 to 1:20
- If the father has CHD, the risk is 1:30

## Indications for fetal echocardiography[6]

### A. Maternal indications

- Diabetes
- Autoimmune disorders like systemic lupus erythematosus (SLE) or Sjogren's syndrome
- Use of drugs, e.g., antiepileptics or antipsychotics like lithium, etc.
- CHD in the mother

### B. Fetal indications:

- History of a sibling with a cardiac anomaly
- Prominent nuchal translucency or increased nuchal fold thickness
- Structural defect in other systems
- Fetal infections
- IUGR in the mid-trimester

### Indications for converting a routine scan into fetal echocardiography

- Chamber asymmetry
- Altered cardiac axis
- Altered position of the fetal heart
- Enlarged fetal heart
- Arrhythmia

## Timing

The fetal heart can be evaluated at any time during the gestation period when USG is done. In the first trimester (11–14 weeks), cardiac details may not be elicited well, but the presence of a pulsatile ductus venosus or tricuspid regurgitation can be a very strong marker for cardiac and chromosomal anomalies.[7]

The fetal heart can be evaluated in the third trimester, but there are a lot of limitations due to oligoamnios and shadowing from the fetal spine, ribs, and limbs.

The best time to evaluate the fetal heart is between 18–22 weeks gestation.[6]

## Equipment

The equipment used for performance of fetal echocardiography needs to have an excellent B-mode, with a good cine-loop facility so that one can scroll back frame by frame and capture the frame of interest. The spatial and temporal resolution needs to be good. The system should have color Doppler, pulsed Doppler, and continuous wave Doppler. STIC, tissue Doppler, and multiplanar imaging are added advantages.

A special pre-set for fetal heart evaluation needs to be created with a high frame rate, decreased persistence, and increased compression. The system should have the ability to zoom the image without causing deterioration of image quality. A higher pulse repetition frequency (PRF) is required for color Doppler in the fetus as compared to the settings used for routine obstetric color Doppler.

## Evaluation of the fetal heart

### A. Position

**Figure 1:**
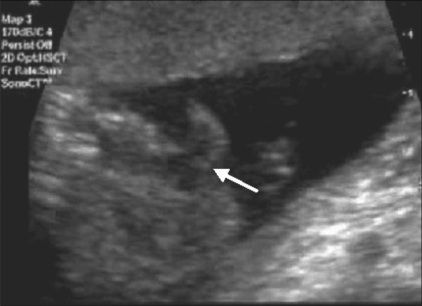
Ectopia cordis. The entire heart (arrow) lies outside the thorax

**Figure 2:**
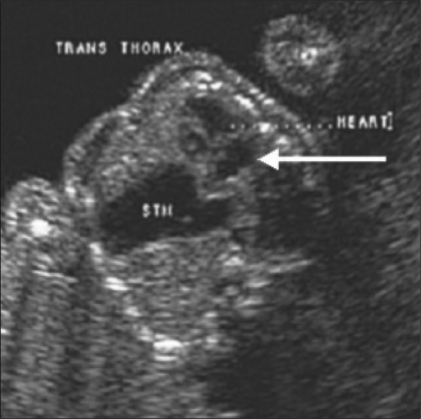
Congenital diaphragmatic hernia. The heart (arrow) is pushed to the right within the thorax and the stomach (STM) is seen in the thorax

The heart may be completely or partially outside the thorax (ectopia cordis) [Figure 1]. The position of the heart may be altered within the thorax, as occurs in congenital diaphragmatic hernia [Figure 2] or pulmonary hypoplasia.

### B. Cardiac axis

**Figure 3:**
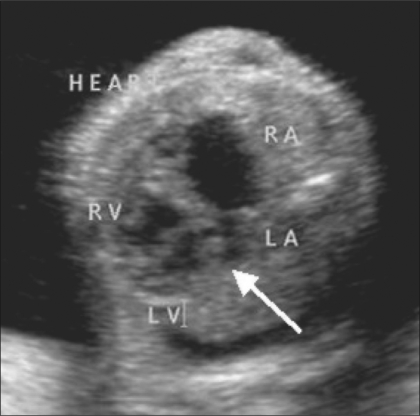
Altered cardiac axis. Left axis deviation of the heart is seen (arrow). RA – right atrium, LA – left atrium, RV – right ventricle, LV – left ventricle

The cardiac axis is the angle the interventricular septum makes with the anteroposterior diameter of the thorax. The normal cardiac axis is 45± 15°. The heart is normally deviated to the left. It is almost entirely within the left chest. An altered cardiac axis is a pointer toward an anomaly. Altered axis is often associated with outflow tract anomalies [Figure 3].

### C. Situs

In order to judge the situs of the fetus, it is essential to figure out which the left side of the fetus is by looking at its presentation and position. After this, one has to confirm that the fetal stomach is to the left. Once the abdominal situs is confirmed as being normal, the transducer is angled cephalad to see if the apex of the heart points to the same side as the stomach.

The normal situs or situs solitus has all the left-sided landmarks, such as the stomach, spleen, the abdominal aorta, and the cardiac apex, to the left of fetus, while the liver and the inferior vena cava (IVC) are to the right of the fetus. A mirror image of this arrangement is called situs inversus. The incidence of cardiac anomalies in the presence of situs inversus is 2%.

**Figure 4:**
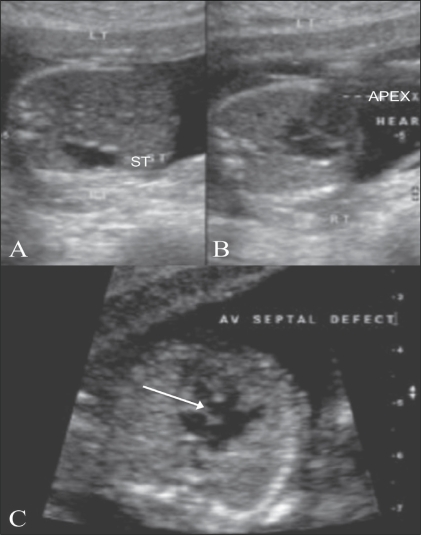
Situs inversus with a cardiac anomaly (A–C). USG image (A) shows the stomach bubble (ST) on the right, while the apex of the heart (APEX) is seen pointing to the left (B). An associated AV septal defect is seen (arrow in C)

Any situs anomaly between these two ends of the spectrum can be labeled as a heterotaxy syndrome. These syndromes have a very high incidence of cardiac anomalies– up to 75–90%. In these patients, it is important to look for an interrupted IVC, presence of an azygos system, a midline liver, and splenic anomalies [Figure 4].

### D. Structural delineation of the fetal heart

There are certain views that *must* be seen and documented, and on each view, there are certain observations that must be made. These views are:

#### 1. Four-chamber view

**Figure 5:**
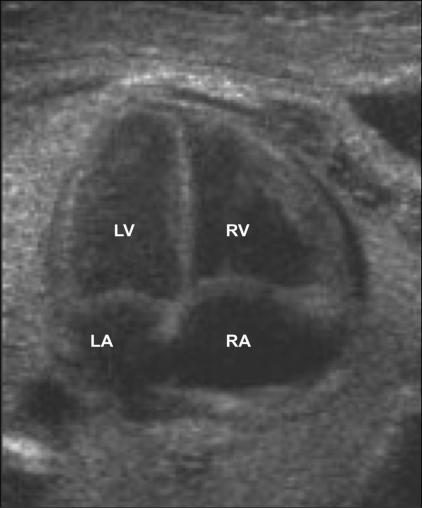
Four-chamber view of the heart: normal appearance. RA-right atrium, RV-right ventricle, LA-left atrium, LV-left ventricl

This view is technically very easy to obtain. It is taken in the transverse section of the thorax. First, a good abdomen perimeter section is obtained; then the probe is slid cephalad to obtain the four-chamber view [Figure 5]. There are three types of four-chamber views: apical, basal, and lateral. The most informative view is the apical or basal four-chamber view. The lateral four-chamber view is the best view to visualize the interventricular and interatrial septum. Necessary observations on the four-chamber view are the following:

- Number of chambers. Normally four chambers are seen.
- Comparison of chamber sizes. Normally both atria are of the same size. The ventricles should also be identical in size, with no evidence of wall thickening.
  a Identification of chambers can be done on the basis of:
  b Position – the chamber closest to the spine is the left atrium and the most anterior chamber is the right ventricle.
- Morphological features – the echogenic moderator band is present in the right ventricle. The flap of the foramen ovale opens into the left atrium.
- The crux of the heart is formed by the membranous part of the ventricular septum, the septum primum of the atrial septum, and the septal leaflets of the mitral and tricuspid valves.
- The septal leaflet of the tricuspid valve is inserted into the septum closer to the apex than that of the mitral valve. This ‘offset’ is normally about 3 mm. Abnormalities of alignment of the valves could be a pointer towards atrioventricular septal defects.
- The ventricular septum has to be examined right from the apex to the crux for any defects. An echo drop-out is often seen in the interventricular septum, especially when the sound beam is parallel to the interventricular septum. In this case, an angle needs to be created between the interventricular septum and the sound beam, upon which this echo drop-out disappears; however, any change due to a ventricular septal defect (VSD) will persist. A true VSD generally has bright margins.
- The two atrioventricular valves—the mitral and tricuspid—should be identified. Opening and closing of these valves has to be evaluated in real time.
- Flow across the atrioventricular valves has to be examined with color Doppler. The direction of flow is from the atria to the ventricles. It is the same across both valves. No aliasing should be seen.
- Area behind the heart: only one vessel, the descending aorta, should be seen between the left atrium and the spine.

### Common anomalies seen on the four-chamber view

**Figure 6:**
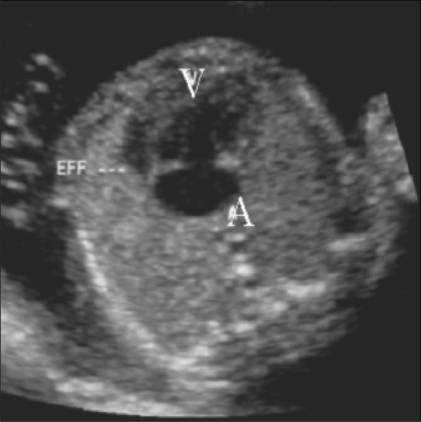
Two-chambered heart. The image shows a single ventricle (V) and a single atrium (A). Note the pericardial effusion (EFF)

**Figure 7 (A–C):**
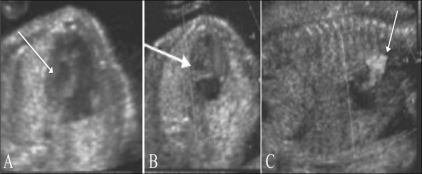
Hypoplastic left heart. USG image (A) shows an echogenic left ventricle (arrow). Doppler images (B,C) show absent flow on the left side (arrow in B), and reversal of flow in the aortic arch (arrow in C)

**Figure 8:**
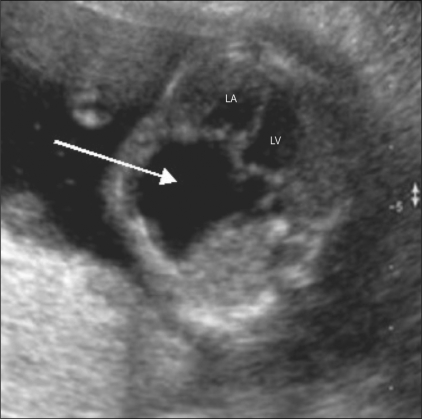
Ebstein's defect. A large right atrium is seen (arrow). RV-right ventricle, LV-left ventricle

**Figure 9:**
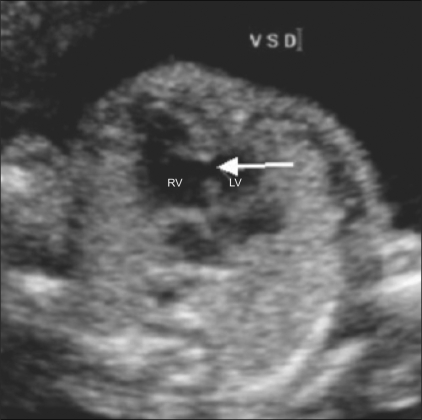
Ventricular septal defect (VSD). A VSD is well seen (arrow) between the right and left ventricles. RV-right ventricle, LV-left ventricle

**Figure 10:**
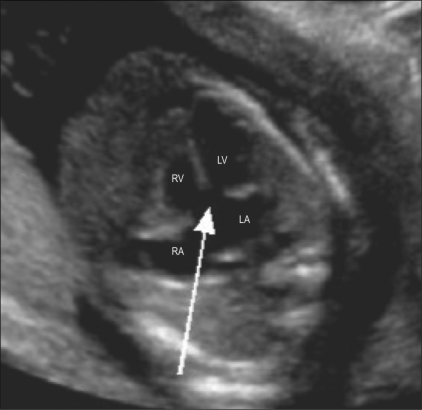
Atrioventricular septal defect. Four-chamber view shows the absent crux (arrow). RA – right atrium, LA – left arium, RV – right ventricle, LV – left ventricle

- Two-chambered heart: This is a defect of septation. The septum that divides the primitive heart into the left and right sides fails to develop. There is a single atrium, a single ventricle, and a single AV valve. This condition is often also associated with a single outflow tract—the truncus. Chromosomal anomalies are known to be associated [Figure 6].
- Hypoplastic left heart [Figure 7]: This is often associated with mitral atresia. The mitral valve is echogenic and does not move during the cardiac cycle. Since there is no flow across the mitral valve, the left ventricle fails to develop normally. The left ventricle is small, echogenic, and does not reveal any color flow. The arch of the aorta reveals reversal of flow, i.e., from the descending aorta into the arch and thence into the ascending aorta. The arch of the aorta fills in a retrograde manner from the ductus.
- Ebstein's anomaly [Figure 8]: This is a progressive disorder. The right atrium is grossly enlarged. The tricuspid valve is more apically placed than usual. Typically, the offset between the mitral and tricuspid valves is around 8 mm. There is regurgitation across the tricuspid valve on color Doppler.
- Ventricular septal defects [Figure 9]: The defect in the ventricular septum has bright margins. There is no flow across the VSD in fetal life because there is no pressure difference between the right and left side of the fetal heart. If the VSD is large, there might be free mixing of blood across the defect.
- Atrioventricular septal defect [Figure 10]: This can be a minor defect like a membranous VSD or a septum primum atrial septal defect (ASD); there could be an absent crux (popularly referred to as endocardial cushion defect); or there could be a single atrioventricular valve. All these structures have a common developmental origin: the endocardial cushion. This defect is often associated with chromosomal anomalies like trisomy 21 and single-gene disorders like Ellis-van Creveld syndrome.

### 2. Three-vessel view [Figure 11]

This view, also, is technically easy to obtain. From the position for a four-chamber view, the probe is slid cephalad for the three-vessel view. The three vessels seen on this view are the pulmonary artery in longitudinal section, seen anteriorly and to the left; the aorta in transverse section, seen in the center; and the superior vena cava (SVC) in transverse section, seen to the right.

**Figure 11:**
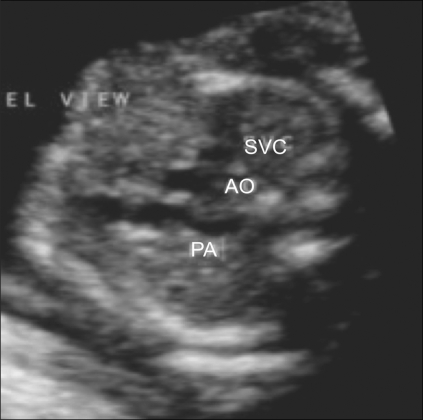
Normal three-vessel view. This image shows a longitudinal view of the pulmonary artery (PA), while the aorta (AO) and superior vena cava (SVC) are seen in cross-section

### 3. Right ventricular outflow tract (RVOT) [Figure 12]

From the position for a four-chamber view, the probe is slid cephalad and rotated towards the left fetal shoulder to obtain the RVOT view. This reveals the RVOT in its long axis. The pulmonary valve motion can be appreciated within the RVOT. On rotation toward the left, the bifurcation of the pulmonary artery can be seen.

**Figure 12 (A–C):**
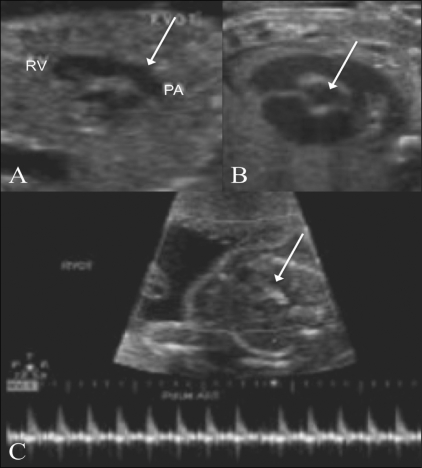
Right ventricular outflow. The pulmonary artery (PA) is seen in long axis (arrow in A) and short axis (arrow in B). Color Doppler (C) image shows color flow and the spectral waveform of the pulmonary artery (arrow). RV – right ventricle

Color and pulsed Doppler evaluation of the RVOT is done to look for aliasing at the pulmonary valve. The peak systolic velocity in the pulmonary artery can be obtained with pulsed Doppler.

### 4. Left ventricular outflow tract (LVOT) [Figure 13]

From the four-chamber view position, the transducer is angled towards the right shoulder of the fetus to visualize the LVOT. The LVOT view can also be opened from the lateral four-chamber view. In this view, 1) the septoaortic continuity needs to be confirmed and 2) the aortic valve motion has to be observed.

**Figure 13 (A,B):**
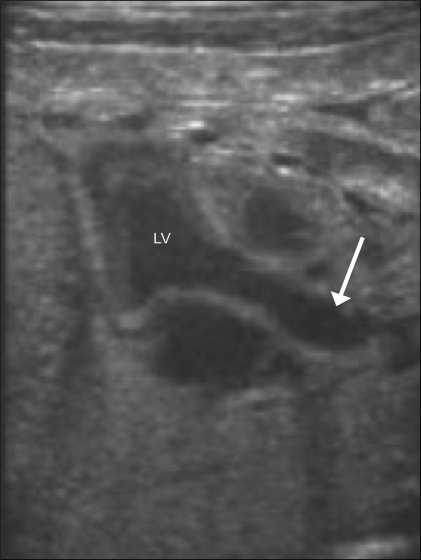
Left ventricular outflow. The aorta is seen in its long axis (arrow), arising from the left ventricle (LV)

The two outflow tracts cross each other at their origins. On color Doppler they reveal opposite color signals at the origin.

### 5. Arch of aorta [Figure 14]

The arch of aorta has to be visualized in a sagittal view. It is narrow and round. The great vessels arise from this arch. On color Doppler, the direction of flow can be seen to be from the ascending aorta to the arch and then to the descending aorta.

**Figure 14 (A,B):**
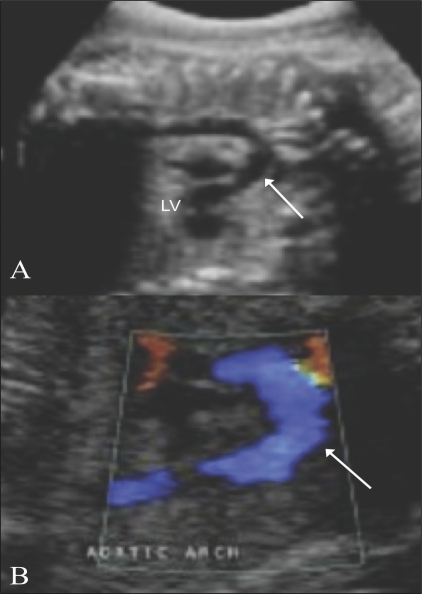
Arch of aorta. Longitudinal views show the aortic arch in B-mode (arrow in A) and with color flow (arrow in B). LV – left ventricle

### 6. Ductal arch [Figure 15]

The ductal arch is flat and wide. It can be seen by angulating the transducer anteriorly from the position for the arch of aorta section. The ductal arch starts anteriorly behind the sternum. On color Doppler, there is an area of aliasing in the arch; this corresponds to the area of the ductus arteriosus. The peak systolic velocity in the ductus arteriosus can be measured with pulsed Doppler.

**Figure 15 (A–C):**
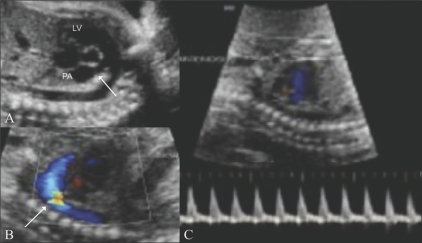
Ductal arch. The ductal arch is seen in B-mode (arrow in A), with color flow (arrow in B), along with its spectral waveform (C). LV – left ventricle. PA – pulmonary artery

### 7. Venoatrial connections [Figure 16]

The venoatrial connections on the right side are visualized on a right parasagittal view. This is popularly known as the ‘hammock view.’ The SVC and IVC are seen to open into the right atrium. On the left side, the pulmonary veins are seen to open into the left atrium. These are best seen in a four-chamber view. Of the four pulmonary veins only two or three may be visualized. On color Doppler, the direction of flow is toward the left atrium.

**Figure 16 (A,B):**
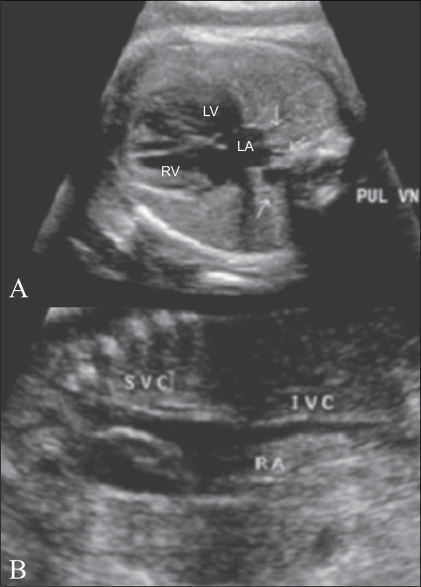
Venoatrial connections. The pulmonary veins (A). The pulmonary veins (PUL VN, arrows) are seen draining into the left atrium (LA). Systemic veins (B). The superior vena cava (SVC) and inferior vena cava (IVC) are seen draining into the right atrium (RA)

## Common anomalies seen on the outflow tract view

### Tetralogy of Fallot [Figure 17]

There is loss of septoaortic continuity. The aorta is seen to override the interventricular septum. There is turbulence at the root of the aorta on color Doppler. The RVOT is relatively narrow and reveals poor or no forward flow.

**Figure 17 (A,B):**
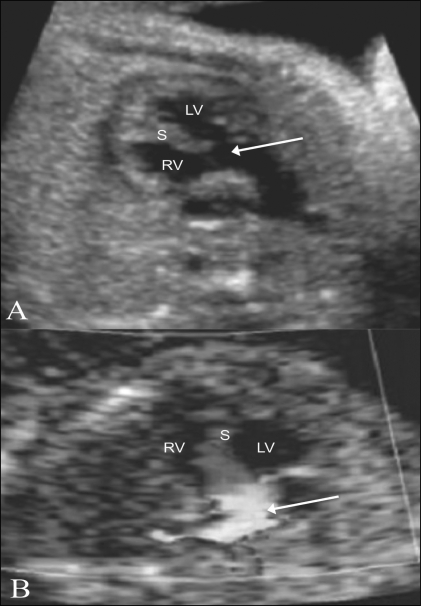
Tetralogy of Fallot. The aorta (arrow) is seen overriding the interventricular septum (S) on this LV outflow view (A), with turbulence (arrowhead) seen on the color flow image (B). RV – right ventricle, LV – left ventricle, S – septum

### Transposition of the great arteries [Figure 18]

Both the outflows are parallel to each other at the origin. They show similar color flows, indicating that the direction of flow in both the great vessels is the same.

**Figure 18 (A,B):**
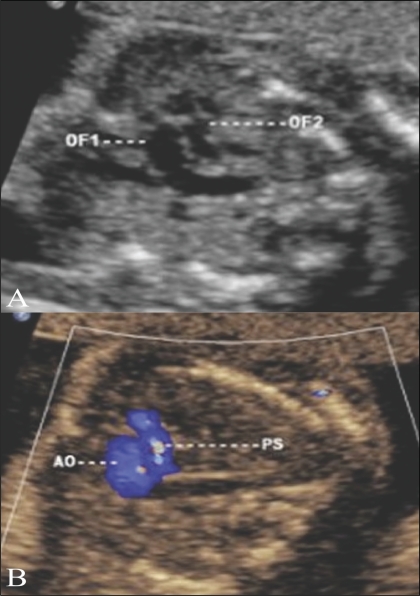
Transposition of the great arteries. An image through both outflow tracts (A) shows the two outflow tracts parallel to each other (OF1, OF2). The color Doppler image (B) shows that the flow in both outflow tracts is in the same direction. Ao – aorta, PS – pulmonary artery.

### Double-outlet right ventricle [Figure 19]

The right ventricle is large and reveals a moderator band. The left ventricle is rudimentary. Both outflow tracts are parallel to each other at the origin and are usually good sized. It is often associated with situs anomalies.

**Figure 19:**
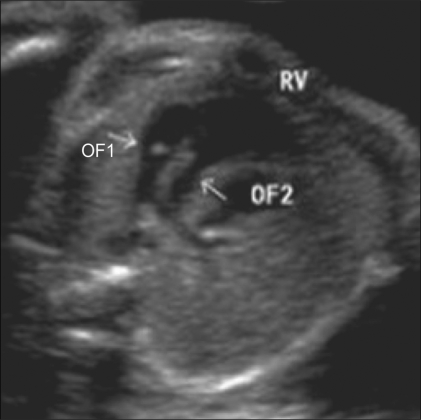
Double-outlet right ventricle. Both outflow tracts (OF1 and OF2) are seen arising from the right ventricle (RV).

### Pulmonary stenosis [Figure 20]

The pulmonary artery is very narrow or may reveal poststenotic dilatation. On real-time echocardiography, the pulmonary valve is not seen to open adequately. On color Doppler, aliasing may be seen. On pulsed Doppler, very high velocities, over 180 cm/s, are noted in the main pulmonary artery.

**Figure 20 (A,B):**
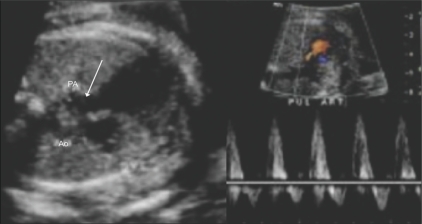
Pulmonary stenosis. A three-vessel view (A) shows pulmonary artery stenosis (arrow); there is a prominent aorta (Ao) since the patient also had a VSD. The color Doppler image (B) shows high peak velocities with aliasing. PA – pulmonary artery.

### E. Rhythm abnormalities of the heart

Rhythm abnormalities are generally picked up by the clinician. The radiologist first needs to rule out a structural anomaly. Then, with the help of M-mode and pulsed Doppler, the rhythm of the heart can be evaluated.

The fetal heart rate varies from 120–160 beats per minute (BPM). A rate below 120 BPM is termed as bradycardia and above 200 BPM is termed as tachycardia. When the fetal heart beat is first visualized at close to 5 weeks of gestation, the rate may sometimes be about 90 BPM, but soon the rate picks up. The most common rhythm abnormality seen in practice is premature atrial contraction followed by a compensatory pause. Heart blocks of varying degrees may be seen. Supraventricular tachycardia is one area where fetal therapy has made a mark; maternal digitalization can make this arrhythmia revert back to normal.

### F. Functional assessment of the fetal heart

Two general points should be assessed:

- Size of the heart: the normal cardiothoracic ratio is 1:2
- Squeeze of the heart: this is most often a visual impression.

**Figure 21:**
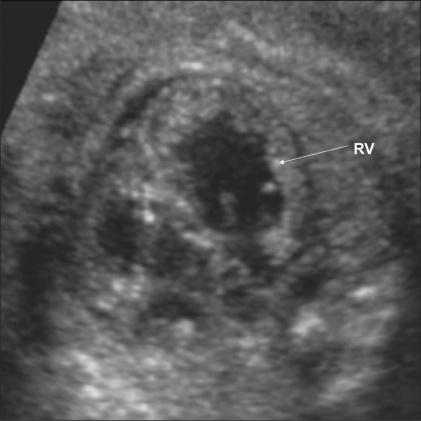
Marked enlargement of the fetal heart is seen due to hypoxia. RV – right ventricle.

Enlargement of the fetal heart and poor contractility are often seen in fetal hypoxia, i.e, in placental insufficiency [Figure 21]. Impaired cardiac function often leads to pericardial effusion.

## Pitfalls

- Some lesions such as minor VSDs may be missed.
- Progressive defects, such as a bicuspid aortic valve, may not be diagnosed at 18–20 weeks of gestation.
- Outflow tract anomalies may be missed.[3]
- Maternal habitus and fetal lie may be limitations.
- Visualization of details may not be possible before 18 weeks.

## Conclusion

To conclude, it may be difficult or time consuming to perform a dedicated fetal echocardiogram on all patients. However, it may be worthwhile looking at the four-chamber view, the outflow tracts, and the three-vessel view; this would be sufficient to diagnose 80–85% of cardiac anomalies.
